# Inhibition of Myeloperoxidase Pro-Fibrotic Effect by Noscapine in Equine Endometrium

**DOI:** 10.3390/ijms24043593

**Published:** 2023-02-10

**Authors:** Ana Amaral, Nélio Cebola, Anna Szóstek-Mioduchowska, Maria Rosa Rebordão, Paweł Kordowitzki, Dariusz Skarzynski, Graça Ferreira-Dias

**Affiliations:** 1 CIISA—Centre for Interdisciplinary Research in Animal Health, Faculty of Veterinary Medicine, University of Lisbon, 1300-477 Lisbon, Portugal; 2 Associate Laboratory for Animal and Veterinary Sciences (AL4AnimalS), 1300-477 Lisbon, Portugal; 3 Department of Zootechnics, School of Sciences and Technology (ECT), University of Évora, 7002-554 Évora, Portugal; 4 Comprehensive Health Research Centre (CHRC), 7000-811 Évora, Portugal; 5 Faculty of Veterinary Medicine, Universidade Lusofona, 1749-024 Lisbon, Portugal; 6 Veterinary Teaching Hospital of the University of Extremadura, 10003 Cáceres, Spain; 7 Institute of Animal Reproduction and Food Research, Polish Academy of Science, 10-748 Olsztyn, Poland; 8 Polytechnic of Coimbra, Coimbra Agriculture School, Bencanta, 3045-601 Coimbra, Portugal; 9 Department of Basic and Preclinical Sciences, Institute for Veterinary Medicine, Nicolaus Copernicus University, ul. Gagarina 1, 87-100 Torun, Poland

**Keywords:** equine, endometrosis, fibrosis, myeloperoxidase, noscapine, inhibition, collagen

## Abstract

Myeloperoxidase is an enzyme released by neutrophils when neutrophil extracellular traps (NETs) are formed. Besides myeloperoxidase activity against pathogens, it was also linked to many diseases, including inflammatory and fibrotic ones. Endometrosis is a fibrotic disease of the mare endometrium, with a large impact on their fertility, where myeloperoxidase was shown to induce fibrosis. Noscapine is an alkaloid with a low toxicity, that has been studied as an anti-cancer drug, and more recently as an anti-fibrotic molecule. This work aims to evaluate noscapine inhibition of collagen type 1 (COL1) induced by myeloperoxidase in equine endometrial explants from follicular and mid-luteal phases, at 24 and 48 h of treatment. The transcription of collagen type 1 alpha 2 chain (*COL1A2*), and COL1 protein relative abundance were evaluated by qPCR and Western blot, respectively. The treatment with myeloperoxidase increased *COL1A2* mRNA transcription and COL1 protein, whereas noscapine was able to reduce this effect with respect to *COL1A2* mRNA transcription, in a time/estrous cycle phase-dependent manner (in explants from the follicular phase, at 24 h of treatment). Our study indicates that noscapine is a promising drug to be considered as an anti-fibrotic molecule to prevent endometrosis development, making noscapine a strong candidate to be applied in future endometrosis therapies.

## 1. Introduction

Neutrophils were mainly described to perform their antimicrobial activity through phagocytosis [1]. Although, in 2004, Brinkmann [2] described a new way of neutrophils fighting pathogens, that was called the neutrophil extracellular traps (NETs). Neutrophil extracellular traps resulted from neutrophil degranulation, followed by the release of DNA filaments and some enzymes, which entrap pathogens, enabling antimicrobial action [2,3,4]. Alongside the NET antimicrobial properties, they have been reported to also contribute to the development of several diseases in many species, such as atherosclerosis, autoimmunity, sepsis, COVID-19, neoplasia, fibrosis, and vascular inflammatory or metabolic diseases [5,6,7,8]. In fact, in our previous studies, we have investigated the pro-fibrotic effect of enzymes present in NETs in equine endometrial explants that can act as pro-fibrotic factors in the equine endometrium [9,10,11,12,13]. Moreover, NETs were recently reported in the airways of a mouse models studying cystic fibrosis in the absence of bacterial infection, revealing their possible role in chronic cystic fibrosis [14]. Additionally, lung fibrosis that results from COVID-19 disease may be due to the excessive production of NETs in these patients [15]. Altogether, these facts make the study of NET involvement in fibrosis quite relevant.

Myeloperoxidase (MPO), elastase and cathepsin G are enzymes released by neutrophils when forming NETs [16,17]. In equine endometrial explants challenged with MPO, it was found that elastase or cathepsin G enzymes could induce collagen type I (COL1) expression in treated explants, linking them to equine endometrial fibrosis development [9,10,11,12,13]. The enzyme MPO is mainly expressed by neutrophils, but it is also expressed by monocytes and macrophages [18]. Its antibacterial action is performed by the production of reactive oxygen intermediates, such as chloramine and hypochlorite produced from the bacteria-induced hydrogen peroxide, which are toxic for bacteria [19,20]. In addition, MPO has been implicated in tissue damage and several inflammatory diseases [18]. The severity of pulmonary lesions in patients with cystic fibrosis is related to the levels of MPO in the sputum [21,22]. The role of MPO on liver fibrosis has also been reported [23]. In fact, MPO has been recently studied as a potential biomarker for chronic diseases previously linked with MPO-derived oxidants [24]. Our previous reports have also shown that MPO-induced COL1 in equine endometrial explants may represent an important endometrosis-triggering factor [9,13]. 

Endometrosis, also known as equine endometrial fibrosis, is a condition that largely affects the reproductive efficiency in this species. It is characterized by fibrotic alterations in a mare’s endometrium, where the normal parenchyma is replaced by collagen fibers [25]. This architectural change involves active or inactive periglandular and/or stromal endometrial fibrosis, with alterations of glands within the fibrotic foci, leading to impaired glandular function [26]. Post-breeding-induced endometritis is a fast physiological mechanism that protects the uterine environment from the contaminating bacteria and debris, but also removes the unnecessary spermatozoa [27,28]. In this process, a large influx of neutrophils arrives at the uterine lumen [29,30]. The breeding-induced endometritis must be solved within 24–48 h. Otherwise, it may lead to a persistent endometritis that, in turn, might trigger endometrosis development [27]. 

Despite the multifactorial causes of this disease, we have been focusing in recent years on the contribution of NET enzymes to endometrosis development [9,10,11,12,13]. In an attempt to pursue the search for endometrosis treatment, we have successfully demonstrated the in vitro capability of some selective inhibitors (sivelestat sodium salt, β-keto-phosphonic acid and 4-aminobenzoic acid hydrazide) to inhibit the pro-fibrotic effect of elastase [11], cathepsin G [12] and MPO [13], respectively. More recently, we have demonstrated that elastase [31] and cathepsin G [32] pro-fibrotic roles on mare endometrial fibrosis was successfully inhibited by noscapine (NOSC). These new findings revealed that it is possible to inhibit both elastase and cathepsin G pro-fibrotic in vitro effects in a non-selective way, using NOSC. These findings open new therapeutic strategies for equine endometrosis.

Noscapine is an alkaloid extracted from poppy, which was earlier reported as an antitussive drug [33,34], with the benefits of not having either nervous or respiratory side effects, nor addictive effects [33]. Besides the NOSC action in a wide range of diseases [35], its anti-neoplasic properties against several types of cancer, in studies carried out in cell lines or in mouse models [34,36,37], are noteworthy. In addition, Kach et al. [38] performed a study that reported NOSC anti-fibrotic properties in vivo in mice and in vitro in pulmonary fibroblasts. Noscapine also showed promising results by improving the treatment efficacy of triple negative breast cancer in a mouse model [39]. 

Thus, we have postulated that MPO-induced COL1 could be inhibited in vitro by NOSC action in equine endometrial explants. The objective was to find a single inhibitor that inhibits more than one NET enzyme, instead of using several selective inhibitors, thus facilitating the therapeutic approach to endometrosis. The aim of this study was to evaluate if the alleged in vitro inhibitory action of NOSC could reduce MPO-induced collagen type I alpha 2 chain (*COL1A2*) mRNA and COL1 protein relative abundance on equine endometrial explants from different estrous cycle phases and times of treatment.

## 2. Results

### 2.1. Viability of Equine Endometrial Explants Data

In order to evaluate explant viability, the lactate dehydrogenase (LDH) viability assay was performed. The principle is that when the cell membrane is damaged, LDH is released to the extracellular compartment. The assay aims to determine the LDH activity using the formula: intracellular LDH activity/total activity (extracellular + intracellular) × 100. The results obtained from LDH viability assay are shown in Table 1. The LDH activity slightly decreased from 1 h to 24 h and 48 h, and was significant between 1 h to 24 h, and 1 h to 48 h (*p* < 0.05). These results were independent of the estrous cycle phase.

### 2.2. The Independent Effect of MPO, NOSC, Time of Treatment and Estrous Cycle Phase, and Their Interaction Combinations

The independent effect of MPO, NOSC, time of treatment and estrous cycle phase on *COL1A2* relative mRNA and COL1 relative protein are listed in Table 2. The results were considered significant at *p* < 0.05. 

### 2.3. COL1 Expression Is Different, Depending on Time of Treatment and the Estrous Cycle Phase

**Figure 1:**
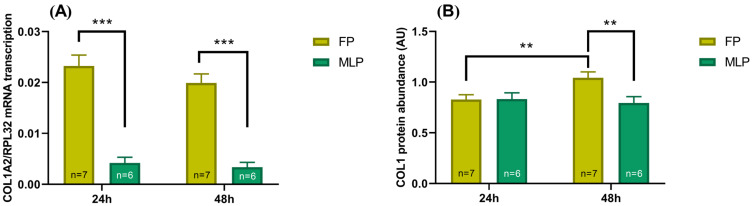
Effect of time of treatment (24 or 48 h) and estrous cycle phase (follicular phase—FP; mid-luteal phase—MLP) on relative collagen type I alpha chain (*COL1A2*) mRNA transcription (**A**), and COL1 relative protein abundance (**B**) in equine endometrial explants. The results are independent of MPO and NOSC treatment, shown as the least square means ± SEM and considered significant at *p* < 0.05. Asterisks above the connecting line represent significant differences between treatments (** *p* < 0.01; *** *p* < 0.001).

The overall effect of time of treatment (24 h or 48 h) and the estrous cycle phase (FP or MLP) was analyzed independently of the MPO and NOSC effect. The results of this interaction are depicted in Figure 1. 

The *COL1A2* mRNA transcription was higher in FP, in comparison to MLP, at both times of treatment (*p* < 0.001; Figure 1A). 

In FP, COL1 relative protein increased at 24 h, with respect to the 48 h treatment (*p* < 0.01; Figure 1B). Moreover, at 48 h of treatment, COL1 relative protein was higher in FP than MLP (*p* < 0.01; Figure 1B).

### 2.4. Noscapine Inhibition on MPO-Induced COL1 Expression Is Dependent on Time of Treatment

**Figure 2:**
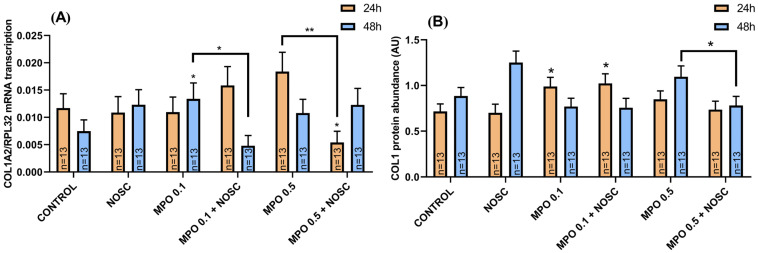
Effect of myeloperoxidase (MPO; 0.1–0.5 μg/mL), noscapine (NOSC; 45 μg/mL), or MPO (0.1–0.5 μg/mL) + NOSC (45 μg/mL) treatments on relative collagen type I alpha 2 chain (*COL1A2*) mRNA transcription (**A**) and collagen type I (COL1) protein relative abundance (**B**) in equine endometrial explants treated for 24 h or 48 h, regardless of the estrous cycle phase. Results are shown as the least square means ± SEM and considered significant at *p* < 0.05. Asterisks represent significant differences relative to the respective control group. Asterisks above the connecting line represent significant differences between treatments (* *p* < 0.05; ** *p* < 0.01).

Myeloperoxidase 0.1 μg/mL treatment in equine endometrial explants provoked an increase in *COL1A2* relative mRNA at 48 h, comparing to control (*p* < 0.05; Figure 2A). However, NOSC reduced this effect with respect to MPO-treated explants (*p* < 0.05; Figure 2A). The MPO 0.1 μg/mL treatment also increased COL1 protein relative abundance at 24 h, which continued to be elevated with MPO 0.1 μg/mL + NOSC treatment, compared to the control group (*p* < 0.05; Figure 2B). At 24 h, NOSC added to MPO 0.5 μg/mL reduced *COL1A2* mRNA with respect to MPO 0.5 μg/mL-treated group (*p* < 0.01; Figure 2A), and to the control group (*p* < 0.05; Figure 2A). The combined treatment of MPO 0.5 μg/mL + NOSC also decreased COL1 protein relative abundance at 48 h, when compared to MPO 0.5 μg/mL-treated group (*p* < 0.05; Figure 2B).

### 2.5. Estrous Cycle Phase Affects the Equine Endometrial Explant Response to MPO and NOSC Treatments

**Figure 3:**
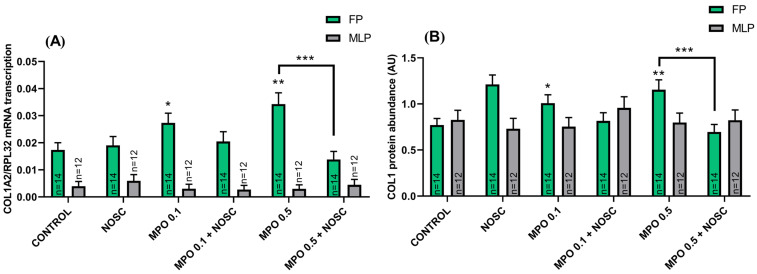
Effect of myeloperoxidase (MPO; 0.1–0.5 μg/mL), noscapine (NOSC; 45 μg/mL), or MPO (0.1–0.5 μg/mL) + NOSC (45 μg/mL) treatments on relative collagen type I alpha 2 chain (*COL1A2*) mRNA transcription (**A**) and collagen type I (COL1) protein relative abundance (**B**) in equine endometrial explants, treated from follicular (FP) or mid-luteal (MLP) phases, regardless of treatment time. Results are shown as the least square means ± SEM and considered significant at *p* < 0.05. Asterisks represent significant differences relative to the respective control group. Asterisks above the connecting line represent significant differences between treatments (* *p* < 0.05; ** *p* < 0.01; *** *p* < 0.001).

In FP, both concentrations of MPO increased COL1 expression (Figure 3). Myeloperoxidase 0.1 μg/mL upregulated *COL1A2* mRNA transcription and COL1 relative protein abundance in FP, relative to the control-treated group (*p* < 0.05; Figure 3A,B). The MPO 0.5 μg/mL treatment also raised *COL1A2* mRNA and COL1 protein, when compared to control (*p* < 0.01; Figure 3A,B), while the addition of NOSC reduced this effect (*p* < 0.001; Figure 3A,B).

### 2.6. NOSC Inhibition of MPO-Induced COL1 Is Dependent on the Time of Treatment and Estrous Cycle Phase

**Figure 4:**
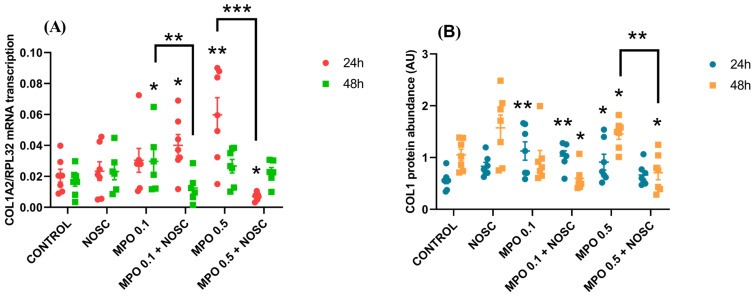
Effect of myeloperoxidase (MPO; 0.1–0.5 µg/mL), noscapine (NOSC; 45 µg/mL), or MPO (0.1–0.5 µg/mL) + NOSC (45 µg/mL) treatments in explants of mare endometrium from the follicular phase (FP; *n* = 7), treated for 24 or 48 h, on relative collagen type I alpha 2 chain (*COL1A2*) mRNA transcription (**A**) and collagen type I (COL1) protein relative abundance (**B**). Results were considered significant at *p* < 0.05 and shown in a scattered dot plot, as the mean ± SEM. Asterisks represent significant differences relative to the respective control and asterisks above connecting lines indicate significant differences of MPO + NOSC treatment relative to the respective MPO-treated group (* *p* < 0.05; ** *p* < 0.01; *** *p* < 0.001).

The *COL1A2* transcription increased in FP at 24 h by MPO 0.5 μg/mL treatment (*p* < 0.01; Figure 4A), and at 48 h by MPO 0.1 μg/mL treatment (*p* < 0.05; Figure 4A), in comparison to control. However, the inhibitory effect of NOSC was detected in both cases, by reducing *COL1A2* mRNA when compared to MPO 0.5 μg/mL and MPO 0.1 μg/mL treatments (*p* < 0.001; *p* < 0.01, respectively; Figure 4A). The *COL1A2* transcription with MPO 0.5 μg/mL + NOSC treatment was also lowered in FP, at 24 h, with respect to control (*p* < 0.05; Figure 4A). 

The COL1 relative protein augmented in FP at 24 h, by the effect of MPO 0.1 μg/mL (*p* < 0.01; Figure 4B; Supplementary Figure S1), MPO 0.1 μg/mL + NOSC (*p* < 0.01; Figure 4B; Supplementary Figure S1) or MPO 0.5 μg/mL (*p* < 0.05; Figure 4B; Supplementary Figure S1). At 48 h, in FP, MPO 0.5 μg/mL increased COL1 protein relative to control (*p* < 0.05; Figure 4B; Supplementary Figure S1), but the addition of NOSC decreased this effect (*p* < 0.01; Figure 4B; Supplementary Figure S1), as MPO 0.5 μg/mL + NOSC also showed lower levels when compared to the control group (*p* < 0.05; Figure 4B; Supplementary Figure S1). Moreover, at 48 h, in FP, MPO 0.1 μg/mL + NOSC treatment decreased COL1 relative protein, with respect to control (*p* < 0.05; Figure 4B; Supplementary Figure S1). 

No differences were found for either MLP-treated explants upon the analysis of *COL1A2* mRNA transcription or for COL1 protein relative abundance.

The list of differences found in the same treatments between the FP and MLP, within each treatment time, is detailed in Supplementary Table S1. The differences found in the same treatments between 24 h and 48 h, and within each estrous cycle phase are shown in Supplementary Table S2. The differences found between the NOSC treatment and the other performed treatments for *COL1A2* transcription and COL1 protein relative abundance data are listed in Supplementary Table S3.

## 3. Discussion

In 2014, Rebordão et al. [40] detected the presence of NETs in the endometria of mares with endometritis. This finding led to new study hypothesis, alluding to the fact that NET persistence in equine endometrium may contribute to endometrosis development once NET enzymes may act as pro-fibrotic agents [9]. Indeed, our previous in vitro studies showed that equine endometrial explants submitted to elastase, cathepsin G or MPO treatments increased COL1 expression [9]. Fibrosis is a degenerative condition where the extracellular matrix deposition is altered, the deposition of COL1 in the lamina propria being one of these alterations [26]. 

In the present study, the overall effect of time of treatment and the estrous cycle phase revealed that *COL1A2* mRNA transcription is higher in FP, both at 24 h and 48 h. Additionally, COL1 protein abundance was raised in FP at 48 h, when compared to 24 h. Under estrogen influence, the typical features of the endometrium in the FP include endometrial thickening, increased muscular tone and vascularization, and a relaxed and open cervix [41]. It is established that estrogen influence contributes to a more reactive endometrium response at estrus, since it provides an inflammatory environment. These results are in accordance with our previous studies about the inhibition of MPO by 4-aminobenzoic acid hydrazide, where equine endometrial explants increased COL1 expression when challenged with MPO treatment only in FP [9,13]. 

This finding reinforces the response variation that occurs due to pro-fibrotic agents, as a function of time of treatment and the estrous cycle phase. This way, our team developed several studies about the inhibition of enzymes present in NETs to understand how their selective and non-selective inhibition would affect COL1 expression in equine endometrial explants.

Beside its anti-microbiological activity, MPO has been largely studied because of its involvement in many diseases, especially in inflammatory diseases. Myeloperoxidase was reported to be a local mediator of tissue damage, that exacerbates inflammatory diseases through the production of reactive oxygen intermediates that injure the surrounding tissues [18]. Additionally, MPO was linked to fibrotic conditions, such as fibrosis induced by nonalcoholic steatohepatitis [42] or lung cystic fibrosis [43]. These findings have provided evidence to support MPO as an important therapeutic target to be considered. Moreover, MPO is considered an important inflammatory indicator of inflammatory responses, especially in the heart and coronary diseases associated with ischemic–reperfusion lesions [44,45]. 

In order to evaluate if the in vitro inhibition of MPO would reduce COL1 expression in equine endometrial explants, we successfully showed that MPO can be inhibited using a selective inhibitor, 4-aminobenzoic acid hydrazide [13]. The selective inhibitors of other enzymes present in NETs, such as elastase and cathepsin G, were also tested and reduced COL1 elastase- and cathepsin G-induced collagen [11,12]. Despite the multifactorial origin of equine endometrosis, finding an inhibitor capable of inhibiting more than one pro-fibrotic factor is imperative and reveals itself challenging. Therefore, we postulated that NOSC would reduce COL1 induced by enzymes present in NETs in a non-selective way. As a matter of fact, NOSC was capable of inhibiting COL1 induced by elastase at both estrous cycle phases and treatment times in equine endometrial explants [31]. In addition, NOSC also inhibited COL 1 induced by cathepsin G, mainly in FP [32]. Our previous results demonstrated that NOSC could act as an anti-fibrotic drug in equine endometrium by inhibiting both elastase and cathepsin G in vitro. To the best of our knowledge, this is the first study demonstrating that NOSC could act as an inhibitor of the MPO pro-fibrotic effect in equine endometrium. Noscapine seems to act as a general inhibitor of enzymes present in NETs, making this molecule a strong candidate to be used in future endometrosis therapies.

When analyzing the effect of MPO, NOSC, estrous cycle and time of treatment together, NOSC inhibited *COL1A2* transcripts induced by MPO 0.1 µg/mL at 48 h and by MPO 0.5 µg/mL at 24 h, in FP. However, the analysis of COL1 protein relative abundance revealed the inhibitory effect was only detected with respect to MPO 0.5 µg/mL at 48 h, in FP. Moreover, both concentrations of MPO increased COL1 protein at 24 h, but NOSC was ineffective in reducing it. In a previous study of ours, the NOSC concentration of 45 µg/mL was only able to reduce the pro-fibrotic effect of elastase [31], while it did not successfully inhibit cathepsin G pro-fibrotic effects, given that it inhibited *COL1A2* mRNA transcription, but not COL1 protein induced by cathepsin G [32]. In fact, NOSC seems to have a dose-dependent effect. The effect of NOSC + tryptophan conjugate in human lung cancer cell lines caused cancer cell death in a dose-dependent manner [46]. Moreover, in breast cancer cell lines, NOSC showed a dose-dependent cytotoxic effect, presumably by the nuclear factor kappa-light-chain-enhancer of an activated B-cell (NF-kβ) pathway inhibition [36]. Protein synthesis is not always directly proportional to the transcription of its gene. One must consider the protein turnover, including its translation and degradation rates, which can vary facing different cellular conditions [47]. Moreover, COL is a complex and stable protein that can take several days to be produced [48]. Therefore, future studies must consider the use of a higher concentration of NOSC to achieve the desirable inhibitory effect of NOSC on MPO-induced COL1. Another interesting approach would be to evaluate the combined treatment with elastase, cathepsin G and MPO, in order to evaluate the extensive effect of NOSC inhibition. This study would also allow for the optimization of NOSC concentration for all of these enzymes present in NETs. However, NOSC may be considered a promising therapeutic tool to use against equine endometrosis in the future. 

Neutrophils are the first response immune cells, which engulf bacteria by the formation of phagosomes. When stimulated, neutrophils activate their nicotinamide adenine dinucleotide phosphate (NADPH)-oxidase complex, that generates superoxide, mainly in the phagosome membrane. Superoxide and neutrophil enzymes, such as MPO, are then released to the phagosome lumen. After, secondary oxidants are also generated by neutrophil MPO. The hydrogen peroxide produced by the neutrophil oxidative burst serves as a substrate for MPO to produce more reactive oxygen species (ROS). This MPO-dependent production of ROS is essential for pathogen killing [49]. However, during this process, MPO is irreversibly inactivated. Additionally, the long staying of pathogens at the sites of inflammation will contribute to the continuous recruitment of neutrophils, which causes tissue damage and has been associated to the pathophysiology of many diseases. In fact, the increased neutrophil ROS production may cause endothelial dysfunction, tissue injury and contribute to chronic inflammation. The high levels of ROS cause damage to the DNA, lipids, and proteins, being capable of disrupting cell membranes, affecting mitochondria and leading to cell death [50].

Recently, in vitro studies about the use of noscapine analogues for use as chemotherapeutic agents against triple negative breast cancer, revealed that the drug caused microtubule disruption and increased ROS levels in tumoral cells [51]. It is known that NOSC causes selective apoptosis only in tumor cells [52]. However, it is not clear how ROS generated by NOSC shows tropism to tumoral cells, nor how it does not show toxicity to healthy cells. Due to this duality between MPO and NOSC in regards to ROS generation, this could be the mechanism that links them in their form of action. Based on current knowledge, one may suggest that NOSC increases ROS levels, which in turn may inactivate MPO, once MPO is disabled by high levels of hydrogen peroxide. 

In ischemia–reperfusion injuries, there is a high production of ROS, causing permanent damage to the tissues, namely, for example, in the brain, heart and kidney (caused by pathological ischemia or organ transplant) [44,45,53]. The generated oxidative burst triggers cell death, characteristic of the ischemia–reperfusion injury [54]. Bradykinin is a peptide released in these injuries, that mediates inflammatory response, vasodilatation and damage to the blood-brain barrier [55]. Therefore, bradykinin, through its receptor B1 and B2, has been linked to mediate the deleterious effects of ischemia–reperfusion [44,53,56]. Interestingly, NOSC has shown protective effects against ischemia–reperfusion injury by blocking bradykinin receptors [44,53]. Noscapine is considered a non-competitive bradykinin receptor antagonist. In fact, it reduced the mortality of stroke patients [57], down-regulated the inflammatory mediators in renal ischemia–reperfusion injury in rats [53] and showed reduction of cerebral damage in ischemia–reperfusion injury in rats [44]. Even so, NOSC mechanism of action in ischemia–reperfusion remains unclear. Notably, NOSC was able to inhibit MPO activity, showed neuroprotective effects, improved cell viability and attenuated neuronal damage in a study of yeasts and ischemia–reperfusion in rats [44]. 

However, some other pathways may mediate the anti-inflammatory or anti-fibrotic action of NOSC. At this level, the inhibition may interfere with general inflammatory/fibrotic pathways that are not targeted directly to the enzymes. The NOSC action mechanism has been mostly studied in cancer. The alkaloid binds to tubulin, causing tumor-cell apoptosis [58]. From the various putative pathways that have been linked to its anti-cancer effect, a study reported that NOSC inhibited the NF-kB pathway [59,60]. This is an important pathway in immune and inflammatory disorders activating pro-inflammatory cytokines [61]. In the case of mares presenting persistent post-breeding endometritis, the pro-inflammatory cytokines remain pathologically upregulated [62]. Persistent post-breeding endometritis is one of the causes of the development of endometrosis [26]. Moreover, in a study conducted by Domino et al. [63], the authors concluded that the gene expression of the NF-kB pathway is altered in endometria that showed signs of fibrosis, but only in FP endometria. These findings suggest that this pathway may be involved in equine endometrosis progression in a hormone-dependent way. Another NOSC action mechanism was reported by Kach [38], where the authors described NOSC anti-fibrotic effects through the prostaglandin receptor E2 (EP2) in human lung fibroblasts. Interestingly, our team provided a study in equine endometrial explants, where EP2 mediated a protective effect against in vitro treatments with enzymes present in NETs [64]. Moreover, the treatment of equine adipose mesenchymal stem cells with prostaglandin (PG)E2, increased the cells’ immunomodulatory competence through EP2 receptor [65]. 

Herein, the authors suggest that the inhibitory effects of NOSC in equine endometrium may be due to the blockade of the NF-kB pathway or through the EP2 receptor. However, further studies must be carried out to unravel the complexity of NOSC action mechanism and elucidate its anti-fibrotic action in equine endometrium. We are aware of some limitations of this study, such as the lack of mechanistic data, which could further elucidate the pathways involved in NOSC inhibition of COL1 induced by MPO in equine endometrial explants. To the best of our knowledge, this is the first preliminary approach to the use of NOSC as future putative therapy for equine endometrosis. In fact, the present data might be the grounds for further investigation of interest for both researchers and veterinary practitioners, who are working in the field of equine endometrosis.

We are looking forward to test NOSC in vivo and confirm its anti-fibrotic action. However, it is important to ensure that NOSC shows no severe side effects, as it has been described in the literature [66]. Nevertheless, animal experimentation must be a very well-reasoned design and has the big limitation of animal handling. Obtaining an adequate number of mares, as well as their handling, requires strict and careful planning. Furthermore, the use of laboratory animal species does not fully suit the equine model, as endometrosis is a specific fibrotic disease of the equine endometrium. Since we have already demonstrated that the response to NET pro-fibrotic enzymes changes according to the estrous cycle phase [9,10,11,12,13], it makes the therapeutic approach to equine endometrosis a complex task. Although anti-fibrotic studies have been pursued in other species and organs, most are valuable to transpose to the equine endometrosis experimental approach. In fact, we do hope that data gathered in our studies on the mare endometrium will contribute to the knowledge on fibrosis in man, as well.

## 4. Materials and Methods

### 4.1. Mares Collection at Abattoir

Mare uteri were collected within 5 min after slaughtering at a local abattoir (Rawicz, Poland), according to the European legislation (EFSA, AHAW/04–027), and under the veterinary official inspection. Only healthy mares were considered for this study. A blood jugular sample was also collected at the abattoir into ethylenediaminetetraacetic acid (EDTA) tubes. Estrous cycle determination was based on ovarian feature observation, and confirmed by progesterone (P4) plasma determinations. The follicular phase (FP) criteria were: plasma P4 concentration < 1 ng/mL and a follicle > 35 mm diameter. The conditions to be considered a mid-luteal phase (MLP) sample were: plasma P4 concentration > 6 ng/mL, follicles between 15 and 20 mm diameter and the presence of a well-developed corps luteum. The uteri that showed the presence of endometritis (increased uterine mucus, altered surface endometrium color and the occurrence of bacteria/neutrophils) were discarded [9,64]. Sample limitation only allowed the use of category IIA and IIB of Kenney and Doig [67], that corresponds to mild to moderate alterations of endometrosis [67]. After collection, uteri from the follicular phase (FP; *n* = 7) and the mid-luteal phase (MLP; *n* = 6) were transported on ice to the laboratory, and immersed in cold Dulbecco’s modified Eagle’s medium (DMEM) F-12 Ham medium (D/F medium; 1:1 (*v*/*v*); D-2960; Sigma-Aldrich, St Louis, MO, USA), supplemented with 100 IU/mL penicillin (P3032; Sigma-Aldrich, St Louis, MO, USA), 100 µg/mL streptomycin (S9137; Sigma-Aldrich, St Louis, MO, USA), and 2 g/mL amphotericin (A2942; Sigma-Aldrich, Burlington, MA, USA). Approximately 80 equids are slaughtered per day, for meat consumption. To perform the several in vitro studies carried out in our laboratory, approximately 10 FP and 10 MLP uteri were collected in each visit to the slaughterhouse. An average of 4 uteri were discarded in each assay due to the presence of signs of endometritis described above, or secondary contamination. 

### 4.2. Equine Endometrial Explant In Vitro Culture

The equine endometrial explants were gathered and prepared, as previously described [11]. In summary, the uteri were washed in phosphate-buffered saline (PBS) with 100 µg/mL streptomycin (S9137; Sigma, St Louis, MO, USA) and 100 IU/mL penicillin (P3032; Sigma, St Louis, MO, USA). To obtain explants of 20–30 mg weight, the strips of endometria were separated from the myometrium using scissors. After, the explants were placed in 24-well cell culture sterile plates (Eppendorf, #0030 722.116) with the culture medium and gentle shaking (150 rpm), for 1 h at 38 °C and 5% CO_2_ in a humidified atmosphere chamber (Biosafe Eco-Integra Biosciences, Chur, Switzerland). Culture media DMEM F-12 was supplemented with antibiotics, antifungal and bovine serum albumin, as described in our previous manuscript [11]. After 1h of pre-incubation, culture media were substituted and the explants treated in quadruplicate for 24 or 48 h, as follows: (i) vehicle (negative control) culture medium; (ii) MPO (0.1 µg/mL or 0.5 µg/mL; orb81997; Biorbyt, Cambridge, UK); (iii) noscapine hydrochloride hydrate (NOSC; 45 µg/mL; N9007; Merck, Darmstadt, Germany); or (iv) MPO (0.1 µg/mL or 0.5 µg/mL) + NOSC (45 µg/mL). Noscapine treatment was added just after the replacement of the culture media, while MPO treatment was added 1 h later. The concentrations of MPO and NOSC were selected based on our previous studies, where MPO 0.1–0.5 µg/mL induced COL1 expression [13], whereas NOSC 45 µg/mL proved to reduce COL1 induced by elastase and cathepsin G [31,32]. After 24 or 48 h of treatment, explants were kept in RNAlater^®^ (R901, Sigma-Aldrich, St Louis, MO, USA) and stored at −80 °C.

### 4.3. Determination of Equine Endometrial Explant Viability

In order to determine the viability of equine endometrial explants, lactate dehydrogenase (LDH) activity was performed using a colorimetric assay kit (ab 102526, Abcam, Cambridge, UK), according to the manufacturer’s instructions and as optimized by our previous study [11]. To assess explant viability, the quotient of the intracellular LDH activity and total activity (extracellular + intracellular) were calculated [68].

### 4.4. Real-Time Polymerase Chain Reaction (qPCR) was Used to Determine COL1A2 mRNA Transcription

The extraction of RNA was performed using the TRI Reagent^®^ (9424; Sigma-Aldrich, St Louis, MO, USA), in accordance with the manufacturer’s protocol. The quantification of RNA was done in a nanodrop system (ND 200C; Fisher Scientific, Hamton, PA, USA). The visualization of bands 28S and 18S allowed for the visualization of RNA quality (1.5% red staining agarose gel; 41,003; Biotium, Hayward, CA, USA). Therefore, cDNA synthesis was achieved using the M-MLV reverse transcriptase enzyme (M5313; Promega, Madison, WI, USA) from 1 µg of total RNA in a 20 µL reaction volume, using an oligo (dT) primer (C1101; Promega, Madison, WI, USA). The primer sequences for target gene *COL1A2* and reference gene ribosomal protein L32 (*RPL32*) are shown in Table 3. Both genes were previously designed and validated using Primer3 Software and Primer Express (Applied Biosystems, Foster City, CA, USA) [9].

For both *COL1A2* and *RPL32* genes, the qPCR reactions were run in duplicate, using a StepOnePlus™ Real-Time PCR System (Applied Biosystems, Warrington, UK) in a 96-well plate (4,306,737; Applied Biosystems, Warrington, UK), and product specificity was analyzed, as previously described [11,69].

### 4.5. Determination of COL1 Protein Relative Abundance by Western Blot Assay

In order to determine COL1 protein relative abundance, a non-staining total protein loading control Western blot was carried out. Total protein extraction (using RIPA buffer) and protein quantification (Bradford reagent) were performed, as previously described [11]. The protein (30 µg) was loaded in 2x Laemmli Loading Buffer and DTT on 8% acrylamide gel (MB04501; Nzytech, Lisbon, Portugal) incorporated with 0.5% (*v*/*v*) 2,2,2-trichloroethanol (808,610; Merck, Darmstadt, Germany). Protein samples and a standard endometrial sample (30 µg, to normalize and compare gels) were run in single lanes and transferred to a nitrocellulose membrane (GE10600001, GE Healthcare, Chicago, IL, USA) [11]. After running, membranes were exposed for 1 min to the UV light (ChemiDoc XRS + System, Bio-Rad, Hercules, CA, USA) to obtain the normalization image. Primary antibody against COL1 (1:1000 diluted; RRID: AB_2891017, 20121, Novotec, Lyon, France) was incubated overnight at 4 °C. The antibody was already validated to equine endometrium [9]. After, the membranes were incubated during 1.5 h at room temperature with the secondary antibody horseradish peroxidase (HRP)-conjugated anti-rabbit (1:20,000; RRID: AB_2617138; P0448, DakoCytomation, Carpinteria, CA, USA). Luminol-enhanced chemiluminescence (Super Signal West Pico, 34077; Thermo Scientific, Waltham, MA, USA) was used to detect COL1 bands in the membranes that were analyzed by the Image Lab 6.0 (Bio-Rad, Hercules, CA, USA) software, using a multichannel protocol. It allowed the detection of total protein lanes in the stain-free total protein membrane image and COL1 bands on the chemiluminescence image [70]. Then, COL1 protein content was calculated by a factor of normalization to adjust for the variability of the loaded protein [70].

### 4.6. Statistical Analysis

The viability data of LDH activity of equine endometrial explants are displayed as the mean ± SEM, considered significant at *p* < 0.05 and were assessed by one-way analysis of variance (ANOVA), followed by a Tukey’s multiple comparisons test (GraphPAD PRISM, Version 6.00, 253 GraphPad Software, San Diego, CA, USA). 

The normality of obtained results was evaluated by visualization and by the Kolmogorov–Smirnov test of Proc Univariate, using SAS v. 9.4 (SAS Institute Inc., Cary, NC, USA). However, the results needed to be converted by the square root procedure, because they did not show a normal distribution. 

The PROC GLM (SAS v. 9.4; SAS Institute Inc., Cary, NC, USA) was used to evaluate *COL1A2* mRNA and COL1 protein relative abundance results from the performed treatments, in a total of 24 combinations (concentration of MPO, effect of NOSC, estrous cycle phase, and time of treatment). The analysis was done in two steps. Firstly, considering the factorial nature of the studied factors, we evaluated the pro-fibrotic factor (MPO: 0, 0.1 and 0.5 µg/mL), the anti-fibrotic factor (NOSC: 0 and 45 µg/mL), the estrous cycle phase (FP and MLP), and time of treatment (24 and 48 h). Secondly, in a total of 24 treatment combinations, we performed an analysis where the response variables were affected by the various treatments considered. Furthermore, we also included in the statistical analysis all the possible two-way, three-way, and four-way interactions. Afterwards, the PDIFF of PROC GLM (SAS v. 9.4; SAS Institute Inc., Cary, NC, USA) compared the least square means of the treatment combinations. The results were significant at *p* < 0.05. Finally, the least square means ± SEM were back-transformed, and graphs were made in GraphPAD PRISM (Version 6.00, 253 GraphPad Software, San Diego, CA, USA).

## 5. Conclusions

Myeloperoxidase is a peroxidase released by neutrophils when forming phagosomes and NETs. However, MPO seems to act as a pro-fibrotic agent in the equine endometrium, once it increased COL1 expression in a hormone- and time-dependent manner. Noscapine is a promising alkaloid to be considered in future use, as an anti-fibrotic drug to prevent endometrosis development. Noscapine inhibited MPO-induced COL1 expression, especially in the follicular phase. Moreover, NOSC was previously reported to inhibit COL1 induced by other NET proteases, such as elastase and cathepsin G. These findings make NOSC a strong candidate to be used in future endometrosis therapies. It seems to act as a general inhibitor, being able to inhibit all the tested enzymes present in NETs in a single way. Nevertheless, the NOSC anti-fibrotic mechanism of action in the equine endometrium should be the subject of further investigation to optimize and validate its therapeutic use for endometrosis. 

## Figures and Tables

**Table 1:** Measurement of lactate dehydrogenase (LDH) activity in equine endometrial explants after 1, 24, or 48 h incubation. Results are presented as the mean ± SEM. Different superscript letters indicate statistical differences within the time of incubation (^a,b^: *p* < 0.05).

Time of Incubation	LDH Activity (%)
1 h	95.19 ± 0.40 ^a^
24 h	90.24 ± 0.70 ^b^
48 h	87.93 ± 0.86 ^b^

**Table 2:** Listed means and levels of significance (*p* values) in the analyses of relative transcript *COL1A2* gene and COL1 protein relative abundance for two- and three-way interactions between the estrous cycle phase, treatment time, and myeloperoxidase (MPO) or noscapine (NOSC) treatments. The results were considered significant at *p* < 0.05.

	*COL1A2* Gene		COL1 Protein	
Factor/Factors Interaction	Mean *(COL1A2/RPL32* mRNA Transcription)	*p* Value	Mean (COL1 Protein Abundance—AU)	*p* Value
MPO	0.000425	0.744	0.007296	0.754
NOSC	0.007189	0.027 *	0.000790	0.862
Time of treatment	0.001246	0.353	0.089686	0.066
Estrous cycle phase	0.196106	<0.00001 ***	0.088325	0.068
MPO × NOSC	0.004735	0.041 *	0.155415	0.0035 **
MPO × time of treatment	0.001531	0.347	0.320149	<0.0001 ***
MPO × estrous cycle phase	0.002762	0.015	0.006394	0.781
NOSC × time of treatment	0.000826	0.450	0.000009	0.985
NOSC × estrous cycle phase	0.007010	0.029 *	0.018122	0.404
Time of treatment × estrous cycle phase	0.000040	0.868	0.137148	0.023 *
MPO × NOSC × time of treatment	0.009824	0.00017 **	0.046136	0.173
MPO × NOSC × estrous cycle phase	0.003486	0.093	0.187116	0.0012 **
MPO × time of treatment × estrous cycle phase	0.001567	0.339	0.053447	0.131
NOSC × time of treatment × estrous cycle phase	0.001222	0.358	0.093944	0.060

* *p* < 0.05; ** *p* < 0.01; *** *p* < 0.001.

**Table 3:** Sequences of primers used for quantitative real-time polymerase chain reaction (qPCR).

Gene (Accession Number)	Sequence 5′-3′	Amplicon
*COL1A2* (XM_001492939.3)	Forward: CAAGGGCATTAGGGGACACA	196
	Reverse: ACCCACACTTCCATCGCTTC	
*RPL32* (XM_001492042.6)	Forward: AGCCATCTACTCGGCGTCA	144
	Reverse: GTCAATGCCTCTGGGTTTCC	

*RPL32*—ribosomal protein L32; *COL1A2*—collagen type I alpha 2 chain.

## Data Availability

Data will be available upon request to the corresponding author.

## Supplementary Materials

The following supporting information can be downloaded at: https://www.mdpi.com/article/10.3390/ijms24043593/s1.
