# Supplementary material for: Inhibition of Myeloperoxidase Pro-Fibrotic Effect by Noscapine in Equine Endometrium
Source: Int J Mol Sci. 2023 Feb 10;24(4):3593. doi: 10.3390/ijms24043593 (PMC9959736; doi:10.3390/ijms24043593)
Supplement: Supplementary file 1 [file ijms-24-03593-s001.zip › ijms-2133299-supplementary.pdf]

Supplementary Materials

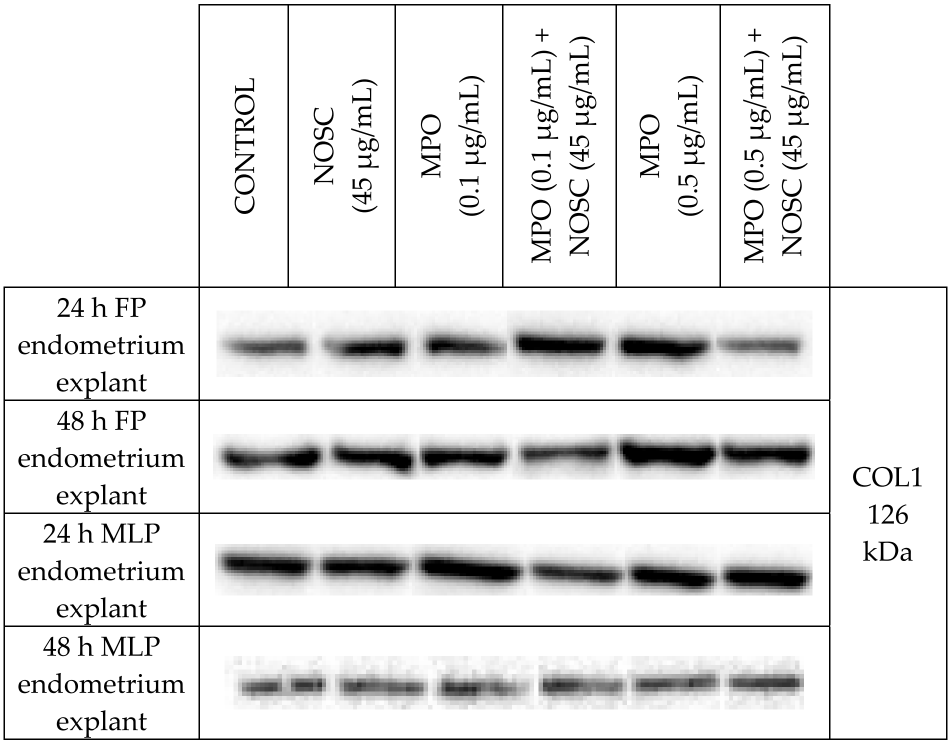

Supplementary Figure S1: Panels representative of type I collagen (COL1) western blotting in follicular phase (FP) and mid-luteal phase (MLP), treated for 24 h or 48 h with myeloperoxidase (MPO; 0.1–0.5 µg/mL), noscapine hydrochloride hydrate (NOSC; 45 µg/mL) and MPO (0.1–0.5 µg/mL) + NOSC (45 µg/mL).

Supplementary Table S1: List of differences found in the same treatments between the follicular phase (FP) and mid-luteal phase (MLP) of the estrous cycle, within each treatment time.

Evaluated Variables	Treatment Comparison	*p*-Value
COL1A2 transcription	MPO 0.1 FP 24h *vs* MPO 0.1 MLP 24h	$p < 0.001$
	MPO 0.1 + NOSC FP 24h *vs* MPO 0.1 + NOSC MLP 24h	$p < 0.001$
	MPO 0.5 FP 24h *vs* MPO 0.5 MLP 24h	$p < 0.001$

COL1A2—collagen type 1 α2; MPO 0.1—myeloperoxidase 0.1 µg/mL; MPO 0.5—myeloperoxidase 0.5 µg/mL; NOSC—noscapine; FP—follicular phase; MLP—mid-luteal phase.

Supplementary Table S2: List of differences found in the same treatments between 24 h and 48 h of treatment, within each estrous cycle phase.

Evaluated Variables	Treatment Comparison	*p*-Value
COL1A2 transcription	MPO 0.1 + NOSC FP 24 h vs. MPO 0.1 + NOSC FP 48 h	$p < 0.01$
	MPO 0.5 FP 24 h vs. MPO 0.5 FP 48 h	$p < 0.05$
	MPO 0.5 + NOSC FP 24 h vs. MPO 0.5 + NOSC FP 48 h	$p < 0.01$
COL1 protein relative abundance	MPO 0.1 + NOSC FP 24 h vs. MPO 0.1 + NOSC FP 48 h	$p < 0.05$
	MPO 0.5 FP 24 h vs. MPO 0.5 FP 48 h	$p < 0.01$

COL1A2—collagen type 1 α2; COL 1 – collagen type I; MPO 0.1—myeloperoxidase 0.1 μg/mL; MPO 0.5—myeloperoxidase 0.5 μg/mL; NOSC—noscapine; FP—follicular phase.

Supplementary Table S3: List of differences found between noscapine (NOSC; 45 μg/mL) treatment, and the other performed treatments: (i) myeloperoxidase (MPO; 0.1 and 0.5 μg/mL) or (ii) MPO (0.1 and 0.5 μg/mL) + NOSC (45 μg/mL) for *COL1A2* transcription and COL1 protein relative abundance in equine endometrial explants from follicular (FP) phase treated for 24 h or 48 h.

Evaluated Variable	Treatment Comparison	*p*-Value	Figure
COL1A2 transcription	NOSC vs MPO 0.1 + NOSC FP 24h	$p < 0.05$	4A
	NOSC vs MPO 0.5 FP 24h	$p < 0.001$	
COL1 protein relative abundance	NOSC vs Control FP 24h	$p < 0.05$	4B
	NOSC vs Control FP 48h	$p < 0.01$	
	NOSC vs MPO 0.1 FP 48h	$p < 0.001$	
	NOSC vs MPO 0.1 + NOSC FP 48h	$p < 0.001$	
	NOSC vs MPO 0.5 + NOSC FP 48h	$p < 0.001$	

COL1A2—collagen type 1 α2; COL 1—collagen type I; MPO 0.1—myeloperoxidase 0.1 μg/mL; MPO 0.5—myeloperoxidase 0.5 μg/mL; NOSC—noscapine; FP—follicular phase.
